# E-Cigarette Users’ Profiles and Their Association with Identified Impacts of COVID-19 on Vaping among Young Adults in Malaysia

**DOI:** 10.3390/healthcare11030434

**Published:** 2023-02-03

**Authors:** Rawaida Mat Salleh, Nizam Baharom, Ching Sin Siau, Caryn Mei Hsien Chan, Noh Amit, Pei Yin Sia, Lei Hum Wee

**Affiliations:** 1 Centre for Community Health Studies (ReaCH), Faculty of Health Sciences, Universiti Kebangsaan Malaysia (UKM), Jalan Raja Muda Abdul Aziz, Kuala Lumpur 50300, Selangor, Malaysia; 2 Primer Care Health Department, Faculty of Medicine and Health Sciences, Universiti Sains Islam Malaysia (USIM), Bandar Baru Nilai, Nilai 71800, Negeri Sembilan, Malaysia; 3 Faculty of Health and Medical Sciences, Taylors University Lakeside Campus, No. 1 Jalan Taylor’s, Subang Jaya 47500, Selangor, Malaysia

**Keywords:** e-cigarette, vaping, COVID-19, young adults, Malaysia

## Abstract

Electronic cigarettes (ECs) users’ profiles and behaviors during the COVID-19 pandemic remain unclear. This cross-sectional study aimed to explore Malaysian EC users’ profiles and their associations with related behaviors during the pandemic. The EC users (*N* = 351) were recruited from an official national vape entity. Respondents were predominantly of Malay ethnicity (90.6%), aged 31 to 35 years (27.6%), males (97.7%), married (68.7%), from Malaysia’s west region states (63.5%) and tertiary educated (69.2%). The majority (80.3%) were non-dual users, and most purchased their vaping products online (77.2%), liked that they can vape while working at home (83.8%) and vaped more because of boredom (55.3%), had low and moderate nicotine addiction levels (94.9%), had low motivation level to quit EC use (92.6%) and were more likely to perceive that vaping did not increase the chances of complications from COVID-19. Respondents with moderate to high addiction levels had twice the odds of checking on their current EC supplies, whilst respondents with low motivation to quit had higher odds of using their tank/pod until the last drop and distancing from others when vaping. EC users should be encouraged to quit EC use, especially during the COVID-19 pandemic.

## 1. Introduction

International estimates found that the prevalence of e-cigarette (EC) use increased from 2.3 million users in 2013 to 5.1 million in 2015 [1]. Among the Southeast Asian countries, Malaysia emerged as a growing EC industry, projecting a million people as regular EC users [2,3]. Malaysia recorded an increase in the prevalence of ECs used among Malaysian adults from 0.8% (15 years old and older) in 2011 [4], 3.2% (18 years and older) in 2016 [5], 4.9% (15 years and older) in 2019 [6] and 5.4% (18 years and older) in 2020 [7].

EC users perceived that ECs have lower toxic effects compared to conventional cigarette smoking [8].

ECs are often preferred over conventional cigarettes because of the cheaper price [9,10], their being perceived as a safer and healthier alternative [11,12] and their greater appeal compared to conventional cigarettes [13]. ECs are claimed to be more acceptable among their peers and close friends than conventional cigarettes [14,15] and are used as tobacco cessation devices. However, adult smokers have been found to use them as an alternative to conventional cigarettes, not as a smoking cessation tool [9,16]. Unmonitored EC use was found to be associated with significantly less quitting among smokers, and most adult EC users continue to additionally smoke conventional cigarettes [17,18]. The effectiveness of the EC as a smoking cessation device is not proven, thus more studies are needed to confirm the degree of effectiveness [10].

By April 2022, the COVID-19 virus had affected more than 506 million people from 226 countries throughout the world and caused more than 6 million deaths worldwide [19]. A study revealed that EC users have a five to seven times greater risk of being infected by COVID-19 [20]. This is because smoking and vaping involve hand-to-mouth contact that may make it easier to spread COVID-19 to the user and other surfaces [21]. Recent data from experimental studies have found that the virus remains stable for several hours to days on surfaces, which makes it plausible for the virus to be transmitted via surfaces of vaping devices [22]. 

During the COVID-19 pandemic, several studies reported that EC use behaviors had changed. For example, a study among 5752 youth and young adults between 15 and 20 years old found that their odds of using ECs decreased after the pandemic began compared to before, and this may be due to restricted access to ECs [23]. However, another study showed that 23% and 41% of EC users reported an increase or no change in EC use after the pandemic began, and those who perceived a higher risk of COVID-19 had three times the odds of quitting EC use [24]. Among US adults who were either cigarette smokers, EC users, or dual users, 27.3% and 23.8% had increased and decreased their EC use during the pandemic, respectively [25]. A national study among adults 16 to 96 years old on consumer risk perceptions of EC use found that more non-EC users perceived that e-cigarette users were more likely to be infected with SARS-CoV-2 compared to EC users, and about 45% had switched from using ECs in a social setting to non-social settings [26]. When asked if they would stop using ECs if they were infected by the COVID-19 virus, 67% said they would whilst 31% said they would continue using [26]. Soule et al. [27] in a study among US residents who used ECs found that several behaviors associated with EC use have changed, including purchasing and procurement of EC supplies, adjustments in EC use frequency and environment and perceptions of health risks in relation to EC use.

Several aspects of EC use in Malaysia were reported in various studies [28,29,30], but little related to the EC users’ profiles and none on related behaviors during the COVID-19 pandemic. Due to the unique challenges posed by the COVID-19 pandemic, this study aimed to explore EC users’ profiles and their associations with related behaviors during the COVID-19 pandemic. Specifically, we aimed to profile EC users based on their age of starting EC use, whether they were daily EC users, single or dual users, frequency of EC use per day, the number of EC puffs per session, reason(s) for using ECs, the source of EC procurement, the number of EC devices possessed, nicotine addiction level and their motivation level to quit ECs. Secondly, we aimed to describe the impact of COVID-19 on their EC use behavior based on the following domains: stocking up and bulk purchasing; challenges in obtaining EC Supplies; alternative purchasing procedures; increased EC use; disruption of routine and EC use; efforts to decrease EC use; improving EC skills; COVID-19 health concerns; perceptions of EC use and COVID-19; and COVID-19 protection. Finally, we aimed to examine the associations between the type of user (dual or non-dual), nicotine addiction level and motivation to quit smoking with various COVID-19 impacts of EC use. Since this is a relatively new area of investigation, we did not put forth any hypotheses.

## 2. Materials and Methods

### 2.1. Study Design, Setting, and Participants

Participants were young adults whose ages ranged from 18 to 44 years old. This online survey is a cross-sectional study that approached Malaysian EC users through the Malaysian Organization of Vape Entity (MOVE) association (https://www.facebook.com/groups/right2vape/, accessed on 17 February 2022), the biggest nationally representative entity that comprised 52,074 registered EC users at the time of the study. EC users were approached through an official online Facebook social media page owned by MOVE. With permission from the president and the administrator of the MOVE Facebook page, a series of announcements were hosted at the Facebook interface between June and August 2021. EC users who wished to participate in the study were required to fill in basic demographic details through an online Google form that consisted of age, gender, e-mail address and telephone contact number.

The inclusion criteria for the sample were Malaysian adults aged 18 years old and above who used ECs. Exclusion criteria were those who refused to provide informed consent, those without Internet access or an electronic device enabling access to the questionnaire and those who did not understand English or Bahasa Malaysia. We estimated the sample size based on a previous study in Malaysia, where 65.3% of EC users expressed their intention to quit [31]. Based on the Cochran formula [32], we assumed a precision *d* of 5%, a confidence interval of 95% and a proportion of 0.653. This resulted in a minimum required sample size of *N* = 350, with a final sample of 385 after adding a 10% non-response rate. Participants were required to fill in the consent form before answering the survey form. The respondents were duly informed that participation in the study was voluntary and that personal identities would remain anonymous. They were also informed that a response to a question was neither ‘right’ nor ‘wrong’. Ethical approval was obtained from the Research Ethics Committee of Universiti Kebangsaan Malaysia (UKM) (approval number: UKM PPI/111/8/JEP-2021-048).

### 2.2. Study Instrument

We adapted a structured, closed-ended, self-administered online questionnaire in English and Bahasa Malaysia based on the current literature [5,33]. The questionnaire was pilot tested among 51 EC users who were not included in the analysis. The purpose of pilot testing the questionnaire was to obtain feedback on the wording of the questions and check if there were irregularities in responses to the questionnaire. Feedback from these EC users during the pilot study helped to rephrase the wording of some questionnaire items. The final questionnaire consisted of 3 sections: (1) sociodemographic background; (2) EC users’ profile; and 3) COVID-19 section. The first section assessed EC user background that included 8 items on sociodemographic characteristics: gender was dichotomized as male or female; age was categorized into age brackets of 18 to 25, 26 to 30, 31 to 35, 36 to 40 or 41 to 44; state was categorized into 4 regions which are north, south, east or west; ethnicity was categorized as Malay, Chinese, Indian and others; marital status was categorized as married, single, divorced or living with partner; education level was categorized as primary education, secondary education or tertiary education; employment status was categorized as private sector, government, self-employed, student or others; and monthly household income. was categorized as the income brackets of <MYR 2000, MYR 2000 to MYR 3999, MYR 4000 to MYR 5999, MYR 6000 to MYR 9999 or ≥MYR 10000 (MYR 1000 was about USD 240).

The second section assessed the EC users’ profiles. The age of starting EC use was assessed by asking participants “What is your age (year) when you first started smoking e-cigarette/vape?”. Daily EC use was assessed using the question “Do you smoke e-cigarette/vape every day or less than every day?” and participants answered either “every day” or “less than every day”. The type of user (dual user or non-dual user) was assessed with the question “Do you currently smoke both e-cigarette/vape and tobacco cigarette?”. Those who answered “yes” were classified as dual users, while those who answered “no”, “don’t know” or “refuse to answer” were classified as non-dual users. Frequency of EC use per day was assessed by the questions “On a WEEKDAY, on average, how many sessions do you usually use e-cigarette/vape?” and “On a WEEKEND, on average, how many sessions do you usually use e-cigarette/vape?”. Participants answered the number of sessions per day, and the sum of the two questions provided the total frequency of EC use in a week. The number of EC puffs per session was assessed using the question “How many puffs do you smoke for each time of e-cigarette/vape use?”. Participants answered with the number of puffs per session. The reasons for using ECs (with yes/no options) were assessed using the question “What is your MAIN reason for smoking e-cigarette/vape? Choose ONE only,” and participants chose from the following options: (1) to quit tobacco cigarette/product; (2) to replace tobacco cigarette/product; (3) to reduce/cut down tobacco cigarette/product used; (4) just to try; (5) to reduce the cost of smoking; (6) to vape in places where tobacco smoking is prohibited; (7) prefer not to respond. The source of EC liquid procurement was assessed by asking the question “Where do you usually buy your e-cigarette/vape liquid?”, and participants were asked to choose one of the following options: online shopping, vape shops, kiosk/shopping center, night market, others and the number of EC devices possessed. This section of the questionnaire was adapted from the National E-Cigarette Survey (NECS) in Malaysia (2016) [5]. The NECS was designed based on input from tobacco control experts from academia and the Ministry of Health Malaysia. This questionnaire was validated among Malaysian adults before a national probabilistic study and was carried out in 2016 [5].

Physical nicotine addiction level was measured using the Fagerström Test for Nicotine Dependence (FTND) adapted for EC use (E-FTND) [34]. It consists of six items that evaluate EC consumption, use compulsion and dependence on ECs. A sample item from the E-FTND is “How many times a day do you vape?” Zero to 1 is used to score yes/no questions, whilst 0 to 3 is used to score multiple-choice items, yielding a total score of 0–10 when all items are summed up. Cut-off scores of 1–3, 4–6 and 7–10 are labeled as low, medium and high nicotine dependence. The internal consistency reliability of the E-FTND in the Malay language was α = 0.725.

Motivation to quit EC use was measured using the Motivation to Stop Scale (MTSS) adapted from cigarette smokers for use among EC users [35]. It consists of one item (“Which of the following describes you?”) with 7 options, ranging from 1 = “I don’t want to stop using e-cigarettes/vape” to 7 = “I REALLY want to stop using e-cigarettes/vape and intend to in the next month.” Higher scores denoted higher motivation to quit EC use. Participants who answered 1 to 5 were grouped as having a low motivation to quit ECs, while those who answered 6 and 7 were grouped as having a high motivation to quit ECs. The MTSS for EC use was back-translated into the Malay language by language and subject-matter experts.

A third section was added to assess COVID-19 in association with EC use, which consists of 20 items adapted from Soule et al. [5] that aimed to evaluate EC user-identified impacts of COVID-19 on vaping during the pandemic. The original questionnaire by Soule et al. [27] was developed using concept mapping based on statements provided by 93 participants on how the COVID-19 pandemic had affected EC use behaviors. Based on the aims of this study and discussions among smoking and EC use experts in Malaysia (LHW, NB, CMHC), 20 items were chosen, 2 items each for the following domains: stocking up and bulk purchasing (e.g., “I check my supplies to make sure I have what I need”); challenges in obtaining EC supplies (e.g., “I go to the store less due to social distancing”); alternative purchasing procedures (e.g., “I already purchase my vaping products online, so I do not need to go out for them”); increased EC use (e.g., “I vape more because I am bored”); disruption of routine and EC use (e.g., “I don’t let anyone else use my vape/EC”); efforts to decrease EC use (e.g., “I am trying to vape less and buy fewer supplies to save money”); improving EC skills (e.g., “I have tried new flavors and brands because of being at home and I don’t have much to do”); COVID-19 health concerns (e.g., “I worry about how vaping is affecting my health”); perceptions of EC use and COVID-19 (e.g., “I am not scared or concerned about vaping due to COVID-19”); and COVID-19 protection (e.g., “I don’t stress too much because I am pretty healthy”). These items had response options of ‘yes’ or ‘no’. The entire questionnaire took about 20 to 30 min to complete.

### 2.3. Statistical Methods

Data collected were analyzed using the IBM SPSS version 28.0. Descriptive statistics were conducted for all variables. Chi-squared test was used to assess the association between the type of EC user and categorical variables in this study. Bivariate analyses were conducted to test the odds ratio of an exclusive EC user reporting the impact of COVID-19 on their EC use. Multiple logistic regression analyses were conducted to examine the association between the impact of COVID-19 on EC use on (1) level of addiction (dichotomized into 0 = low and 1 = moderate and high addiction level); and (2) motivation to stop EC use (0 = high motivation and 1 = low motivation). The level of significance was set at *p* < 0.05 with a 95% confidence interval (*CI*).

## 3. Results

### 3.1. Participants’ Sociodemographic Characteristics

A total of 351 participants responded to the online survey. There were no missing data as answering each question was required. Table 1 shows the participants’ backgrounds. A total of 351 EC users participated in the survey. The mean (*SD*) age of EC users was 33.1 (13.5) years, and the majority were aged 31 to 35 years (*n* = 97; 27.6%). Most respondents were males (*n* = 343; 97.7%), Malays (*n* = 318; 90.6%), married (*n* = 241; 68.7%), had attained tertiary education (*n* = 243; 69.2%), from west region states (*n* = 223; 63.5%) and employed in the private sector (*n* = 168; 47.9%) with a monthly household income ranging between MYR 2000 and MYR 3999 (USD 466 to 932) (*n* = 152; 43.3%).

### 3.2. EC User’s Profile

The mean (*SD*) age of initiation for EC use was 26.8 (5.86) years. Most users used ECs every day (*n* = 310; 88.3%) and were single users, which means they used ECs only (*n* = 282; 80.3%). Most of the users reported the frequency of EC use per day was less than 20 times (*n* = 271; 78.3%), while the mean (SD) number of puffs per session was 19.24 (26.58) puffs. The most popular reason for using ECs was to quit tobacco cigarettes or products (*n* = 219; 62.4%). Most of the users obtained the ECs from vape shops (*n* = 217; 61.8%) and had ≥ 4 devices (*n* = 240; 68.4%) with the mean (SD) 9.77 (12.02). The majority of EC users (*n* = 333; 94.9%) had either a low (*n* = 168; 47.9%) or moderate (*n* = 165; 47.0%) nicotine addiction level. Most of the EC users had low motivation levels to quit ECs (*n* = 325; 92.6%) (Table 2).

### 3.3. EC User-Identified Impacts of COVID-19 on Vaping

Table 3 shows EC user-identified impacts of COVID-19 on vaping. The majority of EC users checked their supplies of ECs to make sure they had what they needed (87.7%), bought extra vaping supplies and e-liquid/pods to stock up (74.1%) and went to the store less due to social distancing (88.0%). However, most of the respondents disagreed that the price of vaping products has increased because of COVID-19 (76.1%). Most of the EC users purchased their vaping products online (77.2%), liked that they can vape while working at home (83.8%) and vaped more because of boredom (55.3%); thus, this situation suggested that they increased EC use during the COVID-19 pandemic. Regarding the disruption of routine and EC use, most of the respondents did not let anyone else use their ECs (86.3%) and tried to distance themselves further from people when using ECs (90.3%). Regarding the effort to decrease EC use, the majority vaped the tanks/pods until the very last drop so they did not have to go out as often (73.5%) and tried to vape less and buy fewer supplies to save money (64.4%). Most of the respondents did not improve their EC skills, had not tried new flavors and brands because of being at home (68.4%) and did not try to perfect their homemade e-liquid flavors because they were at home more (92.0%). Most of the respondents were less likely to worry about the health consequences of vaping (65.5%) and did not think about quitting or reducing their vape usage because of COVID-19 (63.2%) (Table 3).

### 3.4. EC User-Identified Impacts of COVID-19 on Vaping by Type of EC User

Table 3 shows the associations between the type of EC user and their perceptions during the COVID-19 pandemic. The odds of being non-dual users were higher among those who checked their supplies to make sure they had what they needed (*OR* = 2.85; 95% *CI* [1.44, 5.66], *p* = 0.002) and went to the store less due to social distancing (*OR* = 2.03; 95% *CI* [0.99, 4.14]; *p* = 0.050). Being a non-dual EC user protected against perceiving that the price of vaping products had not increased because of COVID-19 (*OR* = 0.55; 95% *CI* [0.31, 0.98], *p* = 0.041), not trying new flavors and brands because of being at home and not having much to do (*OR* = 0.52; 95% *CI* [0.31, 0.90]; *p* = 0.018), not perfecting their homemade or DIY e-liquid flavors because they had more time at home (*OR* = 0.29; 95% *CI* [0.13, 0.64], *p* = 0.001), being less likely to be worried about how vaping can affect their health (*OR* = 0.43; 95% *CI* [0.25, 0.73], *p* = 0.002) and from perceiving that vaping may not kill the COVID-19 virus due to the heat from vaping (*OR* = 0.38; 95% *CI* [0.17, 0.84], *p* = 0.014). (Table 3).

### 3.5. Association between EC Users’ Nicotine Addiction Level, Motivation to Quit ECs and Identified Impacts of COVID-19 on Vaping

Chi-square tests of independence were performed to examine the bivariate association between (1) nicotine addiction level and impacts of COVID-19 on vaping and (2) motivation to quit ECs and impacts of COVID-19 on vaping. Participants with moderate or high nicotine addiction were more likely to report checking their EC supplies to make sure they had what they needed, they liked that they could vape while working from home, they vaped the tanks/pods until the very last drop so they did not have to go out as often, and they tried new flavors and brands because of being at home and they did not have much to do (*p* < 0.05). Multiple logistic regression showed that only checking supplies to make sure they had what they needed was significantly associated with higher odds of moderate to high addiction level (adjusted *OR* = 2.195; 95%*CI* [1.062,4.534], *p* = 0.034). To examine the bivariate association between motivation to quit ECs with COVID-19 impacts on smoking, bivariate analyses showed that those with low motivation to quit were more likely to buy extra vaping supplies and e-liquid/pods to stock up, go to the store less due to social distancing, like that they can vape while working from home, try to distance themselves further from people while they vape, vape the tanks/pods until the last so they do not have to go out as often, are not scared or concerned about vaping due to COVID-19 and are less likely to worry about how vaping was affecting their health (<0.05). Multiple logistic regression showed that only vaping the tanks/pods until the very last drop (adjusted *OR* = 2.852; 95%*CI* [1.163,6.992], *p* = 0.022) and distancing from others while vaping (adjusted *OR* = 3.488; 95% *CI* [1.191,10.215], *p* = 0.023) were associated with higher odds of having low motivation to quit (Table 4 and Table 5).

## 4. Discussion

This study aimed to investigate EC users’ profiles during the COVID-19 pandemic in Malaysia and identify the impact of COVID-19 on EC use behaviors. The key findings were that a large majority of the respondents were non-dual users, were daily EC users, had low and moderate nicotine addiction levels and had low motivation to quit ECs. In the adjusted regression models, those with moderate to high addiction levels had higher odds of checking whether they had enough EC supply, whilst those with low motivation to quit had higher odds of distancing from others when using ECs and completely finishing the tank/pod to avoid going out more often.

This study revealed that the majority of the respondents had low and moderate nicotine addiction levels (94.9%). This finding was consistent with studies by González et al. [36], Etter et al. [37] and Liu et al. [38] which suggested that EC users are less dependent on nicotine than tobacco cigarette smokers. However, studies by Jankowski and colleagues [39] revealed that EC users had higher nicotine dependence levels compared to cigarette smokers. The other studies had younger participants [40,41] compared to this study with an average age of 33.1 (13.5) years, thus suggesting that the patterns of EC use by young adults differ from those of older populations of EC users [42,43]. Furthermore, adolescents and young adults (under 25 years) are also at the greatest risk of nicotine addiction [44].

Most of the EC users purchased their vaping products online (77.2%), preferred to vape while working at home (83.8%) and vaped more because of boredom (55.3%); thus, this situation suggested that they switched their mode of obtaining and had changed their pattern of using ECs or had increased EC use during the COVID-19 pandemic in order to cope with boredom. According to Bommele et al., smokers and EC users appear to be affected by stress related to the COVID-19 pandemic in different ways, either increasing cigarette and EC use or vice versa [45].

From this study, non-dual EC users were more likely to check their supplies to make sure they had what they needed during the COVID-19 pandemic. According to Adriaens et al., although the vape shops closed during the lockdown, EC users in Belgium were able to purchase e-liquid online if it was needed even though online sale of the consumable was banned. This implied that the ban on consumables purchased online is not enforced in Belgium [46] as well as in Malaysia. Thus, for those who were ex-smokers, this situation is a way to avoid smoking relapse [47]. This study showed that the majority of EC users did not perfect their homemade (do-it-yourself; DIY) e-liquid flavors because they had more time at home and perceived that the price of vaping products had not increased because of COVID-19. This suggested that the EC users did not experience financial burden associated with using ECs as the price of the supplies remained unchanged despite the COVID-19 pandemic. Thus, this finding is consistent with a study by Cox et al. which revealed that one of the main reasons for DIY e-liquid is economic and financial savings [48].

The odds of users who had a low motivation level to stop EC use and moderate or high nicotine addiction levels were higher among those who checked the supplies to make sure they had what they needed. According to Jahnel et al., EC users with lower income and education are associated with higher nicotine dependence [49] which consequently may be associated with low motivation to quit ECs. Moreover, it may be that low-income users perceive more stress and use ECs as a coping strategy. Thus, this situation prevented them from being both motivated to stop and attempting to stop using ECs [49]. Findings from the current study were also in accordance with an earlier report in the United Kingdom, which revealed that only one-third of EC users were motivated to stop EC use due to COVID-19 [50].

The results of the multiple regression suggested that overall, participants with moderate to high nicotine addiction and low motivation to quit adjusted their behaviors to the pandemic in order to maintain their EC use. For example, keeping a distance from others, using the tank or pod to the last drop and checking their current EC supplies may allow them to continue their EC use while minimizing their risk for infection. A study by Soule et al. [27] found that those who reported higher EC dependency-rated behavior changes, such as checking on EC stock and taking safety measures when using ECs during the COVID-19 pandemic to be more true of themselves, than those who were not dependent on ECs. The authors suggested that these behavior adaptations allowed them to maintain or obtain EC use behaviors while adhering to the lockdown or social distancing requirements [33].

Regarding the profile of EC users, this current study disclosed that almost three-quarters of the EC users (72.9%) had low monthly incomes (below MYR 4000) and were from the bottom 40% (B40) income group in Malaysia, which earns MYR 4850 or less monthly. This finding is consistent with studies in Malaysia [29,51], the United States [52] and the United Kingdom [53]. Past studies showed that lower socioeconomic status (SES) was an important predictor of conventional smoking among adolescents [54,55]. Green et al. revealed that adolescents from low SES households reported increased initiation and upsurge of tobacco use. Smokers from the lower SES group also reported a lower motivation to quit [56]. Recent research [57,58,59] postulated that the socialization of adolescents from lower-income families, such as the unhealthy lifestyle and health beliefs, could be passed on intergenerationally to influence adolescent smoking behavior.

The majority used ECs daily (88.3%) compared to a previous report by Sapru et al. [60] and the NECS [5] which daily use at only 40% and 26.9%, respectively. Most of the respondents were non-dual users who used ECs only (80.3%) and produced 19.24 mean puffs per session, which was higher compared to the NECS [5] (16.2 puffs per session). Soule et al. suggested that an e-cigarette use session could be variable depending on some factors, such as user, device type and characteristics and situation [39]. Puffing topography is also determined by type of inhalation, such as mouth-to-lung or direct-to-lung inhalation, use of initial clearing puffs, blow back puffs, position in mouth, angle of vaping and any other observed behaviors [61].

Most of the participants claimed to use ECs in order to quit smoking tobacco cigarettes (62.4%) and that their source of ECs was vape shops (61.8%). According to Shi et al. [62], EC use to aid quitting cigarette smoking was not associated with improved cessation or with reduced consumption among early adopters of ECs or even among heavy smokers in the United States. This statement was consistent with a systematic review and meta-analysis by Kalkhoran and Glantz [18] who outlined 20 studies and revealed that cigarette smokers who used ECs as a smoking cessation tool were 28 % less likely to quit smoking compared to those who did not use ECs.

One of the strengths of this study is the participation of EC users from all states in Malaysia, thus improving the internal and external validity of the findings. To the best of our knowledge, this is the first study that explored the identified impacts of the COVID-19 pandemic on vaping among EC users in Malaysia; thus, this provides a plan of action upon which future strategic interventions including public policies could be formulated.

The participants in this survey required access to online computer technology, and thus our recruitment path may have been weighed not only towards those in contact with active EC-use peers, but also those of higher socioeconomic status. However, when using online questionnaires, there is a high possibility of low response rate. The use of incentives, particularly monetary incentives, increases response rates to all survey modes [63]. Due to the cross-sectional nature of the study, any potential causal relationships need to be explained with caution. As is the reality for most questionnaire-based studies, there was no biological verification of self-reported EC-use status.

Despite these limitations, we believe that the aims of this research have been achieved by identifying novel information on identified impacts of COVID-19 on vaping, thus highlighting the need for further studies to better describe the EC users’ related behavior during this pandemic, especially in strategizing the interventions and developing policies to end this vaping epidemic in the future.

## 5. Conclusions

The COVID-19 pandemic period could be the best time for EC users to quit EC use. Past literature shows low success rates to quit nicotine addiction. Facts on health dangers and health risks of vaping linked to COVID-19 should be more actively spread among the public to increase the motivation to quit. Health promotion, prevention and intervention programs for EC cessation are needed to increase the awareness and motivation to quit among EC users, especially young adults, to achieve the *Smoke Free Generation 2045* target [64]. Public health policy makers as well as healthcare professionals can use risk factors in the form of characteristics and perceptions identified in our study findings to help estimate the potential public health impact of EC use, particularly in the context of the COVID-19 pandemic aftermath.

## Figures and Tables

**Table 1:** Sociodemographic characteristics of EC users (*n* = 351).

Sociodemographic Characteristics	Frequency (n)	Percentage (%)
Age (in years)		
Mean (SD)		33.1 (13.5)
18–25	48	13.7
26–30	68	19.4
31–35	97	27.6
36–40	90	25.6
41–44	48	13.7
Gender		
Male	343	97.7
Female	8	2.3
Marital status		
Married	241	68.7
Single	99	28.2
Divorced	9	2.6
Living with partner	2	0.6
Ethnicity		
Malay	318	90.6
Chinese	10	2.8
Indian	2	0.6
Others	21	6.0
Educational background		
Primary	6	1.7
Secondary	102	29.1
Tertiary	243	69.2
Regional area in Malaysia		
West	223	63.5
East	46	13.1
Sabah & Sarawak	36	10.3
South	26	7.4
North	20	5.7
Employment status		
Private sector	168	47.9
Self-employed	92	26.2
Government	62	17.7
Student	14	4.0
Others	15	4.3
Income *		
<MYR 2000	104	29.6
MYR 2000 to MYR 3999	152	43.3
MYR 4000 to MYR 5999	58	16.5
MYR 6000 to MYR 9999	19	5.4
>MYR10000	18	5.1

* MYR 1 = USD 0.24 as of 3 March 2022.

**Table 2:** EC users’ profile (*N* = 351).

Profile	Frequency (*n*)	Percentage (%)
Age started ECs		
Mean (*SD*) in years	26.8 (5.86)	
Daily EC user	310	88.3
Type of EC user		
Non-dual user	282	80.3
Dual user (cigarettes and ECs)	69	19.7
Frequency of EC use per day Mean (*SD*)	9.77 (12.02)	
<20 times/day	271	77.2
≥20 times/day	75	21.4
Did not respond	5	1.4
Number of EC puffs per session		
Mean (*SD*)	19.24 (26.58)	
Reason for using ECs		
To quit tobacco cigarette/product	219	62.4
To replace tobacco cigarette/product	61	17.4
To reduce/cut down tobacco cigarette/product used	39	11.1
Just to try	13	3.7
To reduce cost of smoking	9	2.6
To vape in places where tobacco smoking is prohibited	3	0.9
Did not respond	7	2.0
Source of ECs		
Vape shops	217	61.8
Online shopping	132	37.6
Night market	2	0.6
Number of EC devices		
Mean (*SD*)	9.77 (12.02)	
Nicotine addiction level		
Low	168	47.9
Moderate	165	47.0
High	18	5.1
Motivation to quit ECs		
Low	325	92.6
High	26	7.4

**Table 3:** EC user-identified impacts of COVID-19 on vaping by type of EC user (*n* = 351).

Statement	Total	Type of EC User		χ^2^	*OR*	95% *CI*	*p*-Value
	*n* (%)	Dual User *n* (%)	Non-Dual User *n* (%)				
Stocking Up and Bulk Purchasing							
I check my supplies to make sure I have what I need							
Yes	308 (87.7)	53 (76.8)	255 (90.4)	9.558	2.85	1.44–5.66	0.002
No †	43 (12.3)	16 (23.2)	27 (9.6)				
I bought extra vaping supplies and e-liquid/pods to stock up							
Yes	260 (74.1)	47 (68.1)	213 (75.5)	1.588	1.45	0.81–2.57	0.208
No †	91 (25.9)	16 (23.2)	69 (24.5)				
Challenges in Obtaining EC Supplies							
I go to the store less due to social distancing							
Yes	309 (88.0)	56 (81.2)	253 (89.7)	3.583	2.03	0.99–4.14	0.050
No †	42 (12.0)	13 (18.8)	29 (10.3)				
The price of vaping products has increased because of the COVID-19							
Yes	84 (23.9)	23 (33.3)	61 (21.6)	4.170	0.55	0.31–0.98	0.041
No †	267 (76.1)	46 (66.7)	221 (78.4)				
Alternative Purchasing Procedures							
I already purchase my vaping products online, so I do not need to go out for them							
Yes	271 (77.2)	54 (78.3)	217 (77.0)	0.054	0.93	0.49–1.75	0.816
No †	80 (22.8)	15 (21.7)	65 (23.0)				
I can only buy vapes/ECs from a convenience/grocery store so my options are limited							
Yes	68 (19.4)	19 (27.5)	49 (17.4)	3.664	0.55	0.30–1.02	0.056
No †	283 (80.6)	50 (72.5)	233 (82.6)				
Increased EC Use							
I like that I can vape while working at home							
Yes	294 (83.8)	58 (84.1)	236 (83.7)	0.006	0.97	0.48–2.00	0.940
No †	57 (16.2)	11 (15.9)	46 (16.3)				
I vape more because I am bored							
Yes	194 (55.3)	43 (62.3)	151 (53.5)	1.726	0.70	0.41–1.20	0.189
No †	157 (44.7)	26 (37.7)	131 (46.5)				
Disruption of Routine and EC Use							
I don’t let anyone else use my vape/EC							
Yes	303 (86.3)	56 (81.2)	247 (87.6)	1.941	1.64	0.81–3.30	0.164
No †	48 (13.7)	13 (18.8)	35 (12.4)				
I try to distance myself further from people when I vape							
Yes	317 (90.3)	64 (92.8)	253 (89.7)	0.585	0.68	0.25–1.83	0.445
No †	34 (9.7)	5 (7.2)	29 (10.3)				
Efforts to Decrease EC Use							
I vape the tanks/pods until the very last drop so I don’t have to go out as often							
Yes	258 (73.5)	50 (72.5)	208 (73.8)	0.048	1.07	0.60–1.93	0.827
No †	93 (26.5)	19 (27.5)	74 (26.2)				
I am trying to vape less and buy fewer supplies to save money							
Yes	226 (64.4)	46 (66.7)	180 (63.8)	0.195	0.88	0.51–1.54	0.659
No †	125 (35.6)	23 (33.3)	102 (36.2)				
Improving EC Skills							
I have tried new flavors and brands because of being at home and I don’t have much to do							
Yes	111 (31.6)	30 (43.5)	81 (28.7)	5.581	0.52	0.31–0.90	0.018
No †	240 (68.4)	39 (56.5)	201 (71.3)				
I have more time to perfect my homemade/DIY e-liquid flavours because I am at home more							
Yes	28 (8.0)	12 (17.4)	16 (5.7)	10.369	0.29	0.13–0.64	0.001
No †	323 (92.0)	57 (82.6)	266 (94.3)				
COVID-19 Health Concerns							
I worry about how vaping is affecting my health							
Yes	121 (34.5)	35 (50.7)	86 (30.5)	10.042	0.43	0.25–0.73	0.002
No †	230 (65.5)	34 (49.3)	196 (69.5)				
I am concerned about vaping increasing the chances of complications from COVID-19							
Yes	133 (37.9)	29 (42.0)	104 (36.9)	0.625	0.81	0.48–1.38	0.429
No †	218 (62.1)	40 (58.0)	178 (63.1)				
Perceptions of EC Use and COVID-19							
I am not scared or concerned about vaping due to COVID-19							
Yes	232 (66.1)	48 (69.6)	184 (65.2)	0.461	0.82	0.47–1.50	0.497
No †	119 (33.9)	21 (30.4)	98 (34.8)				
I have thought about quitting or reducing my vaping because of COVID-19							
Yes	129 (36.8)	32 (46.4)	97 (34.4)	3.423	0.61	0.36–1.03	0.064
No †	222 (63.2)	37 (53.6)	185 (65.6)				
COVID-19 Protection							
I don’t stress too much because I am pretty healthy							
Yes	255 (72.6)	46 (66.7)	209 (74.1)	1.547	1.43	0.81–2.52	0.214
No †	96 (27.4)	23 (33.3)	73 (25.9)				
I think vaping may kill the COVID-19 virus due to the heat from vaping							
Yes	30 (8.5)	11 (15.9)	19 (6.7)	6.009	0.38	0.17–0.84	0.014
No †	321 (91.5)	58 (84.1)	263 (93.3)				

† Reference Group.

**Table 4:** Association between EC users’ nicotine addiction level, motivation to quit E-cigarettes and identified impacts of COVID-19 on vaping (*N* = 351).

Statement	Nicotine Addiction Level		*p*-Value	Motivation to Quit ECs		*p*-Value
	Low *n* (%)	Moderate or High *n* (%)		Low *n* (%)	High *n* (%)	
Stocking Up and Bulk Purchasing						
I check my supplies to make sure I have what I need						
Yes	138 (82.1)	170 (92.9)	0.002	288 (88.6)	20 (76.9)	0.080
No	30 (17.9)	13 (7.1)		37 (11.4)	6 (23.1)	
I bought extra vaping supplies and e-liquid/pods to stock up						
Yes	117 (69.6)	143 (78.1)	0.070	245 (75.4)	15 (57.7)	0.048
No	51 (30.4)	40 (21.9)		80 (24.6)	11 (40.3)	
Challenges in Obtaining EC Supplies						
I go to the store less due to social distancing						
Yes	143 (85.1)	166 (90.7)	0.107	290 (89.2)	19 (73.1)	0.015
No	25 (14.9)	17 (9.3)		35 (10.8)	7 (26.9)	
The price of vaping products has increased because of the COVID-19						
Yes	39 (23.2)	84 (23.9)	0.763	76 (23.4)	8 (21.7)	0.396
No	129 (76.8)	267 (76.1)		249 (76.6)	18 (70.9)	
Alternative Purchasing Procedures						
I already purchase my vaping products online, so I do not need to go out for them						
Yes	128 (76.2)	143 (78.1)	0.663	252 (77.5)	19 (73.1)	0.602
No	40 (23.8)	40 (21.9)		73 (22.5)	7 (26.9)	
I can only buy vapes/ECs from a convenience/grocery store so my options are limited						
Yes	27 (16.1)	68 (19.4)	0.134	63 (19.4)	5 (80.8)	0.985
No	141 (83.9)	282 (80.6)		262 (80.6)	21 (19.2)	
Increased EC Use						
I like that I can vape while working at home						
Yes	132 (78.6)	162 (88.5)	0.012	277 (85.2)	17 (65.4)	0.008
No	36 (21.4)	21 (11.5)		48 (14.8)	9 (34.6)	
I vape more because I am bored						
Yes	86 (51.2)	108 (59.0)	0.141	184 (56.6)	10 (38.5)	0.073
No	82 (48.8)	75 (41.0)		144 (43.4)	16 (61.5)	
Disruption of Routine and EC Use						
I don’t let anyone else use my vape/EC						
Yes	144 (85.7)	159 (41.0)	0.750	282 (86.8)	21 (80.8)	0.392
No	24 (14.3)	24 (13.1)		43 (13.2)	5 (19.2)	
I try to distance myself further from people when I vape						
Yes	150 (89.3)	167 (91.3)	0.533	298 (91.7)	19 (73.1)	0.002
No	18 (10.7)	16 (8.7)		27 (8.3)	7 (26.9)	
Efforts To Decrease EC Use						
I vape the tanks/pods until the very last drop so I don’t have to go out as often						
Yes	113 (67.3)	145 (79.2)	0.011	246 (75.7)	12 (46.2)	0.001
No	55 (32.7)	38 (20.8)		79 (24.3)	14 (53.8)	
I am trying to vape less and buy fewer supplies to save money						
Yes	108 (64.3)	118 (64.5)	0.970	211 (64.9)	15 (57.7)	0.459
No	60 (35.7)	65 (35.5)		114 (35.1)	11 (42.3)	
Improving EC Skills						
I have tried new flavours and brands because of being at home and I don’t have much to do						
Yes	46 (27.4)	65 (35.5)	0.033	103 (31.7)	8 (30.8)	0.922
No	122 (72.6)	118 (64.5)		222 (68.3)	18 (69.2)	
I have more time to perfect my homemade/DIY e-liquid flavours because I am at home more						
Yes	8 (4.8)	20 (10.9)	0.120	26 (8.0)	2 (7.7)	0.956
No	160 (95.2)	163 (89.1)		299 (92.0)	24 (92.3)	
COVID-19 Health Concerns						
I worry about how vaping is affecting my health						
Yes	51 (30.4)	70 (38.3)	0.213	106 (32.6)	15 (57.7)	0.010
No	117 (69.6)	113 (61.7)		219 (67.4)	11 (42.3)	
I am concerned about vaping increasing the chances of complications from COVID-19						
Yes	58 (34.5)	75 (41.0)	0.504	122 (37.5)	11 (42.3)	0.630
No	110 (65.5)	108 (59.0)		203 (62.5)	15 (57.7)	
Perceptions of EC Use and COVID-19						
I am not scared or concerned about vaping due to COVID-19						
Yes	114 (67.9)	118 (64.5)	0.869	222 (68.3)	10 (38.5)	0.002
No	54 (32.1)	65 (35.5)		103 (31.7)	16 (61.5)	
I have thought about quitting or reducing my vaping because of COVID-19						
Yes	61 (36.3)	68 (17.4)	0.869	115 (35.4)	14 (53.8)	0.060
No	107 (63.7)	115 (30.5)		210 (64.6)	12 (46.2)	
COVID-19 Protection						
I don’t stress too much because I am pretty healthy						
Yes	121 (72.0)	134 (73.2)	0.801	238 (73.2)	17 (65.4)	0.388
No	47 (28.0)	49 (26.8)		87 (26.8)	9 (34.6)	
I think vaping may kill the COVID-19 virus due to the heat from vaping						
Yes	10 (6.0)	20 (10.9)	0.096	29 (8.9)	1 (3.8)	0.373
No	158 (94.0)	163 (89.1)		296 (91.1)	25 (96.2)	

**Table 5:** Multiple logistic regression of identified impacts of COVID-19 on vaping.

Statement ^†^	Nicotine Addiction Level ^a^		*p*-Value	Motivation to Quit ECs ^b^		*p*-Value
	a*OR*	95% *CI*		a*OR*	95% *CI*	
Constant	0.247		0.001	0.614		0.531
I check my supplies to make sure I have what I need	2.195	1.062–4.534	0.034			
I like that I can vape while working at home	1.540	0.824–2.880	0.176	1.522	0.550–4.209	0.419
I vape the tanks/pods until the very last drop so I don’t have to go out as often	1.585	0.963–2.606	0.070	2.852	1.163–6.992	0.022
I have tried new flavours and brands because of being at home and I don’t have much to do	1.314	0.824–2.097	0.252			
I bought extra vaping supplies and e-liquid/pods to stock up				1.205	0.467–3.111	0.700
I go to the store less due to social distancing				2.362	0.797–7.002	0.121
I try to distance myself further from people when I vape				3.488	1.191–10.215	0.023
I worry about how vaping is affecting my health				0.440	0.183–1.057	0.066
I am not scared or concerned about vaping due to COVID-19				2.380	0.989–5.730	0.053

^†^ Reference group = No. ^a^ 0 = low addiction level; 1 = moderate to high addiction level; Chi square (4) = 17.33; *p* = 0.002; Nagelkerke *R*^2^ = 0.06; ^b^ 0 = high motivation to stop vaping; 1 = low motivation to stop vaping; Chi square (7) = 31.98; *p* < 0.001; Nagelkerke *R*^2^ = 0.020.

## Data Availability

The data that support the findings of this study are available from the corresponding author upon reasonable request.
